# High miR-200a-3p expression has high diagnostic values for hypertensive disorders complicating pregnancy and predicts adverse pregnancy outcomes

**DOI:** 10.1186/s12884-022-04785-x

**Published:** 2022-06-15

**Authors:** Xin He, Danni Ding

**Affiliations:** Department of Obstetrics, Hunan Provincial People’s Hospital, The First Affiliated Hospital of Hunan Normal University, 61 Jiefang West Road, Changsha, 410005 Hunan China

**Keywords:** Hypertensive disorders complicating pregnancy, miR-200a-3p, Diagnosis, Receiver operating characteristic curve, Prognosis, Gestation hypertension, Mild preeclampsia, Severe preeclampsia

## Abstract

**Background:**

Hypertensive disorders complicating pregnancy (HDCP) are various heterogeneous conditions. microRNA (miR)-200a-3p is involved in HDCP diagnosis. This study explored the effects of miR-200a-3p on HDCP patients.

**Methods:**

A total of 126 singleton HDCP patients including 50 cases of gestation hypertension (GH), 42 cases of mild preeclampsia (MP) and 34 cases of severe preeclampsia (SP), were enrolled as study subjects, and 50 normal pregnant women were selected as the control. Serum miR-200a-3p expression was detected and its efficacy in HDCP diagnosis and grading was evaluated. GH, MP and SP patients were allocated to high/low miR-200a-3p expression groups. The correlation between miR-200a-3p expression and general clinical indexes was analyzed. HDCP patients were allocated to high/low miR-200a-3p expression group and maternal and fetal outcomes were followed up. Effects of miR-200a-3p expression on adverse pregnancy outcome incidence were analyzed.

**Results:**

miR-200a-3p expression in the serum of HDCP patients was upregulated. The sensitivity and specificity of serum miR-200a-3p level > 1.201 were 87.3% and 96.0%, respectively. Serum miR-200a-3p level in GH, MP and SP patients was increased with the aggravation of the disease. The cut-off value and area under the curve (AUC) of miR-200a-3p for GH, MP and SP diagnosis were 1.145 and 0.9094 (82.0% sensitivity and 88.0% specificity), 1.541 and 0.8126 (73.8% sensitivity and 76.0% specificity), and 1.866 and 0.7367 (64.7% sensitivity and 76.2% specificity), respectively. Serum miR-200a-3p level was correlated with general clinical indexes, fetal birth weight, systolic to diastolic ratio, and fetal growth restriction incidence. High serum miR-200a-3p expression in HDCP patients was associated with increased adverse pregnancy outcomes.

**Conclusion:**

High miR-200a-3p expression could help to diagnose HDCP, judge severity and was associated with increased adverse pregnancy outcomes.

## Introduction

Hypertension is a prevalent clinical condition in pregnancy, which may have short-term and long-term risks of complications for both infants and mothers [1]. Hypertensive disorders complicating pregnancy (HDCP) are a heterogeneous series of conditions including gestational hypertension (GH) and preeclampsia [2]. HDCP is estimated to influence 3–10% of the pregnant women, with the first pregnancy-young women at the greatest risk [3]. A nephrolithiasis history results in a higher risk of HDCP, especially for the women who are with high body mass index (BMI) during the early pregnancy [4]. HDCP patients have twice the risk of cardiovascular disease as those with normal blood pressure during pregnancy [5]. HDCP is also related to impaired insulin resistance and glucose tolerance and a three to fourfold elevated risk of diabetes [4]. Due to the severe adverse effects of the disease, it is of vital importance to establish the therapeutic methods and search for candidate molecules as the potential biomarkers of HDCP [6].

microRNAs (miRNAs) are a series of non-coding RNA transcripts with a length of about 22 nucleotides, which provide principal post-transcriptional regulation for gene expression in disease and health by sequence-specific binding to the region of 3’-untranslated of the target mRNA transcripts [7]. miRNAs are vital regulators for the cellular functions fundamental for the processes of healthy pregnancy, such as trophoblast cell angiogenesis and differentiation, which are deregulated in pregnancy complications pathogenesis, including preeclampsia [8]. The various abnormally expressed miRNAs are associated with adverse pregnancy outcomes, and the researchers are making efforts to study biological effect of placental miRNAs that contribution to HDCP [9]. miR-200a is reported to directly target and inhibit the EG-VEGF expression to block placental trophoblast invasion in preeclampsia, which is identified as a promising therapeutic target and miRNA biomarker for HDCP [10]. In addition, miR-200a-3p participates in the trophoblast function regulation [11]. However, the expression of miR-200a-3p in the serum of HDCP patients and the relationship between them are still unclear. There is little domestic and foreign report at present on miR-200a-3p expression and clinical research in serum of patients with HDCP. The relationship between the expression of miR-200a-3p and the diagnosis, disease condition and prognosis of HDCP needs to be further explored. This study investigated the role of serum miR-200a-3p expression in the diagnosis of HDCP and its relationship with pregnancy outcomes, so as to provide certain reference value for the diagnosis and prognosis of HDCP.

## Materials and methods

### Ethics statement

The experiments were authorized by the academic ethics committee of Hunan Provincial People’s Hospital, The First Affiliated Hospital of Hunan Normal University. All procedures were strictly implemented by the code of ethics. All the subjects involved were fully informed of the objective of the study and signed informed consent before sampling. The study was in accordance with the relevant guidelines and regulation.

### Study subjects

A total of 126 pregnant women in late singleton pregnancy with HDCP who were treated in Hunan Provincial People’s Hospital, The First Affiliated Hospital of Hunan Normal University from April 2018 to April 2021 were prospectively selected as the research group (HDCP). The patients were assigned to the following 3 subgroups according to the severity of the disease: gestational hypertension group (GH, *N* = 50), mild preeclampsia group (MP, *N* = 42), and severe preeclampsia group (SP, *N* = 34). The 50 normal pregnant women in late singleton pregnancy of the same period were selected as the negative control group (NC). The first day of diagnosis was taken as the baseline data registration time, and all pregnant women were followed up until delivery. The whole blood samples and 24-h urine samples of enrolled pregnant women were collected. The sampling work was carried out by two nurses who had no knowledge of this study.

### Diagnostic, inclusion and exclusion criteria

The diagnostic criteria of HDCP were in accordance with the Diagnosis and treatment guideline of hypertensive disorders in pregnancy (2015) published by the Obstetrics and Gynecology Subcommittee of the Chinese Medical Association in 2015. GH was defined by blood pressure (BP) ≥ 140/90 mmHg after 20 weeks of gestation and returned to normal at 12 weeks postpartum, urine protein (-). MP was defined by BP ≥ 140/90 mmHg after 20 weeks of gestation, urine protein ≥ 0.3 g/24 h or random urine protein ( +) accompanied by the symptoms of abdominal discomfort or headache. SP was defined by that on the basis of MP, any of the following complications existed: BP ≥ 160/110 mmHg, urine protein ≥ 2.0 g/24 h or random urine protein (+ +), serum creatinine (S-Cr) > 106 umol/L, platelet (PLT) < 100 × 10^9^/L, increased serum alanine aminotransferase (ALT) or aspartate transaminase (AST), persistent epigastric discomfort, persistent headache or other neurological or visual disorders. The normal pregnant women were in late singleton pregnancy (≥ 28 weeks) with normal blood and urine routine, liver and kidney function during pregnancy without complications during pregnancy and before and after delivery.

Exclusion criteria were as follows: chronic hypertension, anemia or other cardiovascular diseases before pregnancy; a history of thyroid disease before pregnancy; multiple pregnancy; severe mental illness or cognitive impairment; diabetes history before pregnancy; severe liver and kidney diseases; other major diseases.

### Data collection and clinical index detection

The following data of the enrolled pregnant women were recorded: age of pregnant women, gestational age (diagnosis enrollment time), gravidity, parity, body mass index (BMI = weight (kg)/square of height (m^2^)) during pregnancy, systolic and diastolic blood pressure. In the morning of the 2^nd^ day after enrollment, 8 mL of fasting elbow vein blood of the subjects was collected and separated into EDTA-K2 anticoagulant vacuum blood collection tubes (YA1291, Solarbio, Beijing, China), with 1 mL sample for PLT count determination within 1 h. The remaining blood was centrifuged at 2000 × g for 10 min and the supernatant was absorbed, separated into the centrifuge tube and stored at -80 °C in a refrigerator for S-Cr, AST, ALT, and lactate dehydrogenase (LDH) determination and total RNA extraction. PLT count solution was purchased from BioRoYee (DA0156, Beijing, China). The concentrations of S-Cr (BS-E4325H1, Boshen biotechnology, Nanjing, Jiangsu, China), LDH (QN-PS0351, Qiaoyubio, Shanghai, China), AST (XG-E99150, Xigebio, Shanghai, China), ALT (CK-E10348, Puyibio, Wuhan, Hubei, China) were detected using the ELISA kits. Protein content in urine was determined using a urine protein quantitative test kit (SNM297-KVE, Biolab, Beijing, China). The fetal umbilical artery systolic to diastolic ratio (S/D) at 34 weeks of gestation was detected by color Doppler ultrasound. Sampling and testing were carried out by three medical staff that was unaware of this study in advance.

### Follow-up of adverse pregnancy outcomes

The median expression of miR-200a-3p was taken as the boundary, and all HDCP patients (126 cases) were assigned into miR-200a-3p low expression group and high expression group. The enrolled patients were followed up until delivery. The information of maternal and fetal outcomes was collected after delivery, and the gestational weeks, fetal weight, incidence of fetal growth restriction (FGR) and pregnancy outcomes were recorded. Adverse pregnancy outcomes were defined as that during follow-up or delivery, any outcome of maternal and fetal including eclampsia, heart failure, pulmonary edema, acute kidney injury, cerebrovascular accident, postpartum hemorrhage, placental abruption, fetal distress, premature birth, neonatal asphyxia and perinatal death existed. Premature delivery was defined as less than 37 weeks at delivery. Neonatal asphyxia was defined as an Apgar score below 7 of 1-min after birth.

### Total RNA extraction and reverse transcription quantitative polymerase chain reaction (RT-qPCR)

The 0.5 mL serum sample was added with TRIzol reagent (YT2188, Yitabio, Beijing, China), placed at room temperature for 5 min, added with 0.2 mL chloroform, mixed well, placed at room temperature for 15 min, and centrifuged at 12,000 g for 5 min and the supernatant was transferred to another centrifuge tube. Subsequently, the total RNA was purified using RNA purification kit (DP412, Tiangenbio, Beijing, China) according to the manufacturer’s instructions. The concentration and purification of extracted RNA was determined using an ultraviolet spectrophotometer and cDNA was synthetized using Omniscript RT Kit (50) (Qiagen205111, QIAGEN, Duesseldorf, Germany). The RT-qPCR was performed using ChamQTM SYBR qRT-PCR MasterMix (Vazyme biotech, Nanjing, Jiangsu, China). The reaction system was as follows: pre-denaturation at 95 °C for 10 min and 40 cycles of denaturation at 95 °C for 10 s, annealing at 60 °C for 20 s and extending at 72 °C for 10 s. The relative expression of miR-200a-3p standardized by the internal reference U6 was calculated using the 2^−ΔΔCt^ method. The primer sequences are shown in Table 1.

**Table 1:** Real-time PCR primer sequence

Gene	Forward 5’-3’	Reverse 5’-3’
*miR-200a-3p*	CGCGTGTAGCAATGGTCTGT	AGTGCAGGGTCCGAGGTATT
*U6*	CTCGCATCGGCAGCACA	AACGCTACTCGAATTGCGT

### Data analysis

SPSS 22.0 statistical software (IBM Corp. Armonk, NY, USA) and GraphPad Prism 8.0 software (GraphPad Software Inc San Diego, CA, USA) were used for data analysis and mapping. Shapiro Wilk was used for the normal distribution test. The measurement data in normal distribution were expressed as mean ± standard deviation. Independent *t* test or Chi-square test was used for comparisons between 2 groups, and one-way analysis of variance (ANOVA) was applied for comparisons among groups. The receiver operating characteristic (ROC) curve was used to evaluate the diagnostic efficiency of miR-200a-3p and obtain the cut off value. The Chi square test and Kaplan–Meier were used to analyze the effects of miR-200a-3p expression on the incidence of adverse pregnancy outcomes. The Log rank method was used to test the differences of Kaplan Meier curve between groups. *P* value was obtained by a bilateral test.* P* < 0.05 was indicative of statistical significance.

## Results

### Clinical baseline characteristics of the subjects

In the subjects of this study, compared with the NC group, in the HDCP group, there were no statistical differences in the age, gestational age, gravidity, parity, and BMI (all *P* > 0.05), which were increased as the severity of the disease. SBP, DBP, S-Cr, AST, ALT, LDH and 24-h urine protein were increased and were increased as the aggravation of the disease, PLT was decreased, the gestational week of delivery was advanced, the fetal weight was decreased, the S/D value was increased, and the incidence of FGR was increased (all *P* < 0.05) (Table 2).

**Table 2:** Comparison of clinical baseline characteristics

Parameters	NC (*N* = 50)	HDCP (*N* = 126)		
—	—	GH (*N* = 50)	MP (*N* = 42)	SP (*N* = 34)
Age (year)	29.2 ± 3.5	28.5 ± 3.2	28.7 ± 3.7	29.1 ± 4.2
Gestational age (week)	32.8 ± 2.4	33.1 ± 1.7	32.5 ± 1.9	33.4 ± 2.3
Gravidity (time)	1.72 ± 0.54	1.70 ± 0.51	1.71 ± 0.55	1.79 ± 0.48
Parity (time)	1.13 ± 0.27	1.08 ± 0.27	1.12 ± 0.30	1.09 ± 0.29
BMI (kg/m^2^)	25.0 ± 2.0	24.9 ± 2.2	25.1 ± 2.2	25.0 ± 2.25
SBP (mmHg)	121.4 ± 9.2	144.9 ± 7.2^a^	159.4 ± 12.2^ab^	175.5 ± 17.7^abc^
DBP (mmHg)	75.1 ± 7.1	96.5 ± 8.8^a^	110.2 ± 10.4^ab^	120.9 ± 12.1^abc^
PLT (× 10^9^/L)	222.1 ± 19.8	165.4 ± 16.2^a^	134.2 ± 13^ab^	89.5 ± 13^abc^
S-Cr (umol/L)	43.4 ± 7.1	67.1 ± 9.7^a^	98.9 ± 11.5^ab^	123.9 ± 9.9^abc^
AST (U/L)	29.3 ± 5.7	47.5 ± 9.1^a^	66.7 ± 8.0^ab^	80.0 ± 9.5^abc^
ALT (U/L)	17.8 ± 3.4	29.2 ± 6.0^a^	47.5 ± 5.8^ab^	59.6 ± 7.4^abc^
LDH (U/L)	143.2 ± 10.4	164.6 ± 11.9^a^	181.6 ± 14.6^ab^	208.9 ± 13.5^abc^
24-h urine protein (g)	0.05 ± 0.01	0.17 ± 0.03^a^	0.50 ± 0.13^ab^	2.88 ± 0.62^abc^
Gestational week of delivery (W)	39.8 ± 0.3	38.6 ± 0.5^a^	37.8 ± 0.6^ab^	37.2 ± 0.4^abc^
Fetal weight (g)	3963 ± 565	3385 ± 486^a^	3016 ± 463^ab^	2664 ± 472^abc^
S/D	2.36 ± 0.32	3.27 ± 0.35^a^	3.96 ± 0.41^ab^	4.52 ± 0.43^abc^
FGR	3 (6%)	9 (18%)^a^	15 (35.7%)^ab^	19 (55.9%)^abc^

*BMI* Body Mass Index, *SBP* Systolic Blood Pressure, *DBP* Diastolic Blood Pressure, *PLT* Platelet count, *S-Cr* Serum Creatinine, *AST* Aspartate Transaminase, *ALT* Alanine Transaminase, *LDH* Lactate Dehydrogenase, *S/D* Fetal umbilical artery Systolic to Diastolic ratio, *FGR* Fetal Growth Restriction

The measurement data were expressed as mean ± standard deviation. One-way ANOVA or Chi-square test was used for comparisons between 2 groups.

^a^ Represented compared with the NC group, *P* < 0.05;

^b^ Represented compared with the GH group, *P* < 0.05;

^c^ Represented compared with the MP group, *P* < 0.05

### miR-200a-3p was upregulated in the serum of HDCP patients

**Fig. 1:**
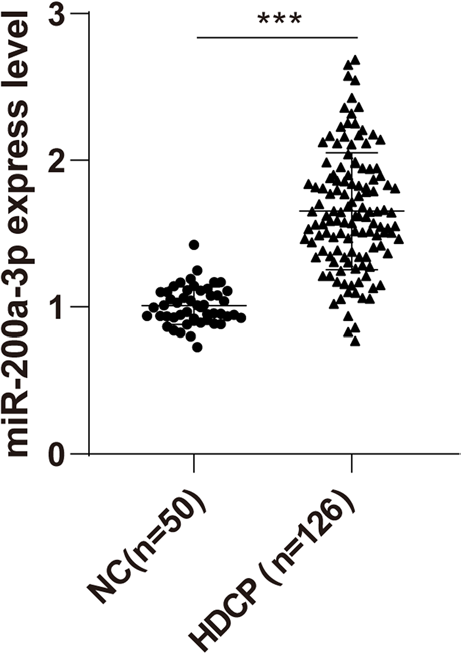
The expression of miR-200a-3p in the serum of HDCP patients. The expression of miR-200a-3p in the serum of the tested pregnant women was detected by RT-qPCR. The data were expressed as mean ± standard deviation. Independent *t* test was used for comparisons between 2 groups. ^***^P < 0.001

The expression of miR-200a-3p in the serum of pregnant women in the NC group and HDCP group was detected by RT-qPCR. The expression of miR-200a-3p in the serum of pregnant women of the NC group was 1.005 ± 0.128 and that of the HDCP group was 1.655 ± 0.379, suggesting that the expression of miR-200a-3p was upregulated in the serum of HDCP pregnant women (Fig. 1, *P* < 0.001).

### miR-200a-3p assisted the diagnosis of HDCP

**Fig. 2:**
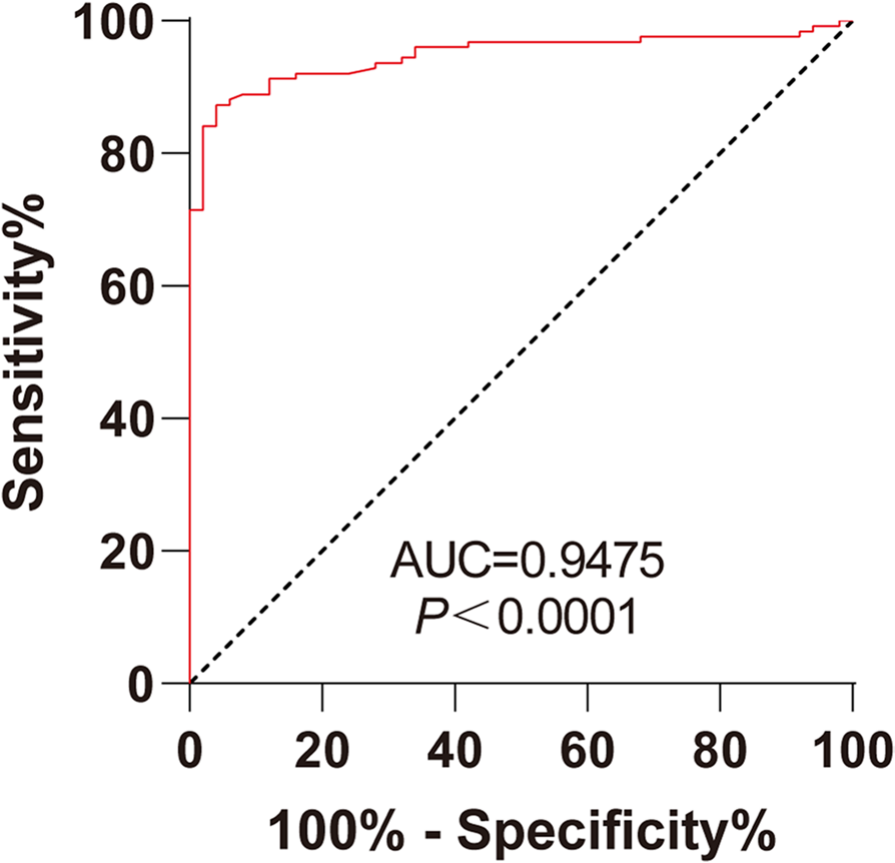
ROC curve of miR-200a-3p in diagnosis for HDCP. ROC curve analysis of miR-200a-3p in the diagnosis for HDCP

The ROC curve of miR-200a-3p expression that distinguished HDCP from normal pregnant women was drawn (Fig. 2). The area under the curve (AUC) was 0.9475 and the cut off value was 1.201 (with the sensitivity of 87.3% and the specificity of 96.0%). These results suggested that serum miR-200a-3p level > 1.201 could assist the diagnosis of HDCP.

### miR-200a-3p is a biomarker of disease severity for HDCP

**Fig. 3:**
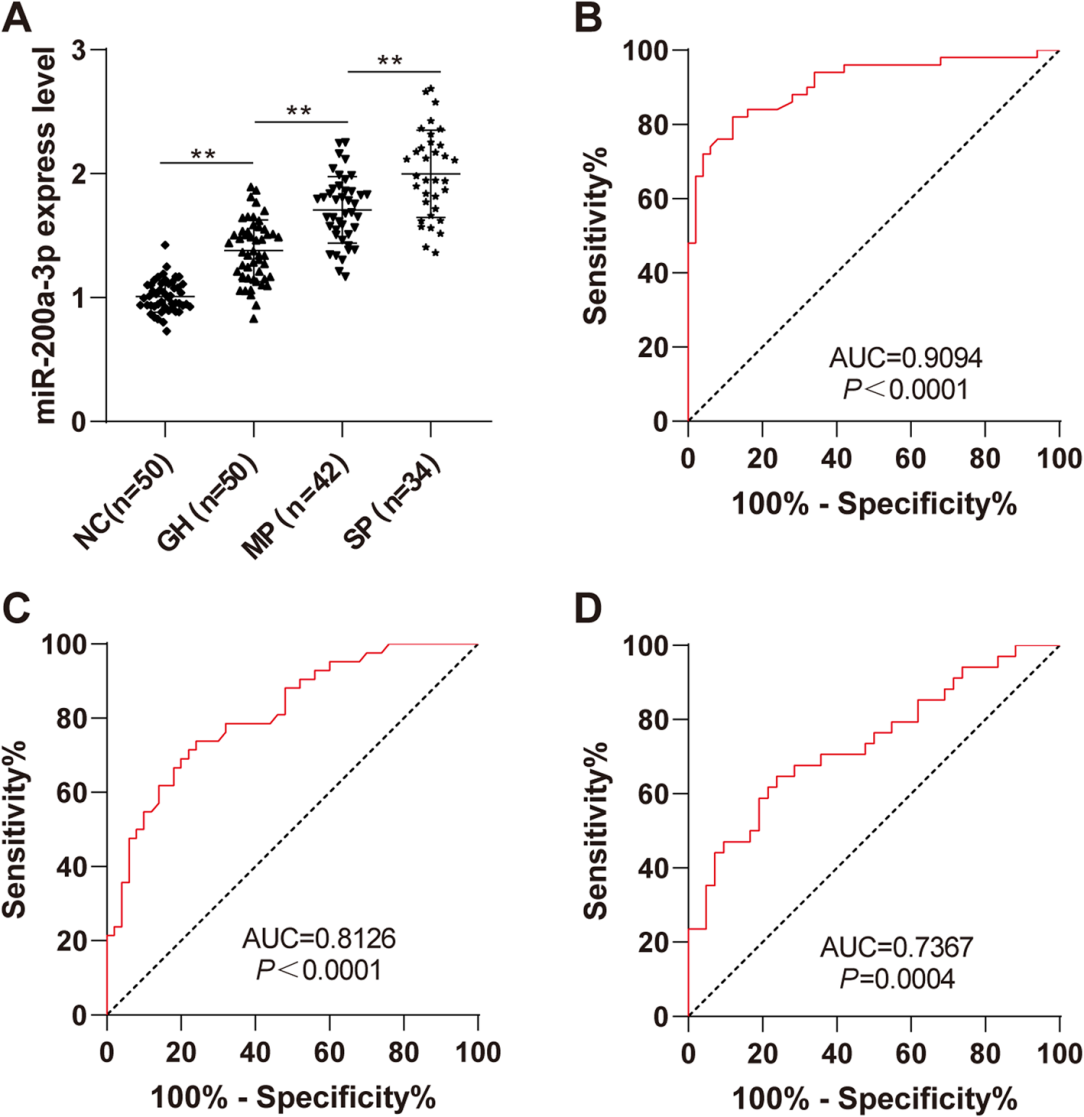
Expression of miR-200a-3p in the subgroups of HDCP. The miR-200a-3p expression in the subgroups of HDCP was detected by RT-qPCR. **A** The expression difference of miR-200a-3p in GH, MP and SP; **B** The ROC curve of miR-200a-3p in diagnosis for GH; **C** The ROC curve of miR-200a-3p in diagnosis for MP; **D** The ROC curve of miR-200a-3p in diagnosis for SP. One-way ANOVA was used for test of panel **A** and ROC was used for analysis of panel (**B**, **C** and **D)**. ^**^P < 0.01

To study whether miR-200a-3p was related to the severity of HDCP, the HDCP group was divided to 3 subgroups including GH group, MP group and SP group. The expressions of miR-200a-3p in the serum of pregnant women in different groups were detected by RT-qPCR and its diagnostic value was analyzed using the ROC curve. Compared with the NC group, the expression of serum miR-200a-3p was significantly upregulated in the GH group (Fig. 3A, *P* < 0.01), its AUC = 0.9094 and the cut-off value was 1.145, with 82.0% sensitivity and 88.0% specificity (Fig. 3B, *P* < 0.0001); compared with the GH group, the expression of serum miR-200a-3p was significantly upregulated in the MP group (*P* < 0.01), its AUC = 0.8126 and the cut-off value was 1.541, with 73.8% sensitivity and 76.0% specificity (Fig. 3C, *P* < 0.0001); compared with the MP group, the expression of serum miR-200a-3p was significantly upregulated in the SP group (*P* < 0.01), its AUC = 0.7367 and the cut-off value was 1.866, with 64.7% sensitivity and 76.2% specificity (Fig. 3D, *P* < 0.0001). These results suggested that miR-200a-3p might be a potential biomarker for HDCP severity classification.

### Correlation analysis of miR-200a-3p and clinical indicators of patients

According to the median value of miR-200a-3p expression, the patients in the GH, MP and SP groups were assigned to low expression group (low EP) and high expression group (high EP), and then the correlation with the clinical indicators of HDCP patients was analyzed. In the GH, MP and SP groups, there were no significant differences in age, gestational age, gravidity, parity between the high EP group and the low EP group; in the GH group, compared with the low EP group, BMI, SBP, DBP and AST were increased, the gestational week was advanced, the fetal weight was decreased, S/D value was increased, and the incidence of FGR was increased in the high EP group (all *P* < 0.05) and there were no significant differences in other indexes; in the MP group, compared with the low EP group, BMI, SBP, DBP, AST and 24-h urinary protein were increased, the gestational week was advanced, fetal weight was decreased, the S/D value was increased, and the incidence of FGR was increased in the high EP group (all *P* < 0.05) and there were no significant differences in other indexes; in the SP group, compared with the low EP group, BMI, SBP, DBP, S-Cr, AST, ALT, LDH and 24-h urinary protein were increased and PLT was decreased, the gestational week was advanced, fetal weight was decreased, the S/D value was increased, and the incidence of FGR was increased in the high EP group (all *P* < 0.05) (Table 3).

**Table 3:** Correlation between serum miR-200a-3p expression and clinical indicators in HDCP patients

Parameters	GH (*N* = 50)		MP (*N* = 42)		SP (*N* = 34)	
	Low EP	High EP	Low EP	High EP	Low EP	High EP
	(*N* = 25)	(*N* = 25)	(*N* = 21)	(*N* = 21)	(*N* = 17)	(*N* = 17)
Age (year)	27.9 ± 3.2	29.2 ± 3.1	28.4 ± 3.8	29.0 ± 3.6	27.8 ± 2.5	28.4 ± 4.1
Gestational week	33.6 ± 2.1	33.1 ± 1.3	32.5 ± 2.3	32.5 ± 1.6	33.6 ± 2.9	33.1 ± 1.5
Gravidity (time)	1.6 ± 0.3	1.8 ± 0.4	1.7 ± 0.6	1.7 ± 0.5	1.9 ± 0.5	1.7 ± 0.5
Parity (time)	1.1 ± 0.3	1.0 ± 0.2	1.1 ± 0.3	1.1 ± 0.3	1.1 ± 0.3	1.1 ± 0.2
BMI (kg/m^2^)	23.3 ± 0.9	26.7 ± 1.3^a^	23.2 ± 1.1	26.7 ± 1.6^b^	23.3 ± 1.1	26.8 ± 1.8^c^
SBP (mmHg)	139.2 ± 4.5	150.5 ± 4.4^a^	149.8 ± 8.2	168.2 ± 7.5^b^	162.0 ± 11.5	189.1 ± 11.2^c^
DBP (mmHg)	89.6 ± 5.4	103.3 ± 5.3^a^	102.0 ± 7.0	117.8 ± 6.4^b^	111.6 ± 7.9	130.2 ± 7.7^c^
PLT (× 10^9^/L)	166.4 ± 17.7	164.4 ± 14.8	135.8 ± 12.8	132.7 ± 13.2	99.5 ± 8.2	79.5 ± 8.5^c^
S-Cr (umol/L)	67.6 ± 9.7	66.7 ± 9.9	100.6 ± 7.5	97.3 ± 13.9	116.4 ± 6.4	131.5 ± 6.3^c^
AST (U/L)	40.4 ± 5.6	54.7 ± 5.5^a^	60.3 ± 5.4	72.5 ± 4.9^b^	72.8 ± 6.1	87.3 ± 6.0^c^
ALT (U/L)	28.8 ± 5.9	29.5 ± 6.2	48.5 ± 6.2	46.6 ± 5.2	54.0 ± 4.8	65.2 ± 4.6^c^
LDH (U/L)	166.4 ± 13.3	162.9 ± 10.3	181.8 ± 15.6	181.4 ± 13.6	198.6 ± 8.8	219.3 ± 8.5^c^
24-h urine protein (g)	0.17 ± 0.03	0.16 ± 0.03	0.50 ± 0.09	0.69 ± 0.08^b^	2.33 ± 0.09	3.42 ± 0.40^c^
Gestational week of delivery (W)	39.0 ± 0.3	38.2 ± 0.3^a^	38.3 ± 0.4	37.4 ± 0.4^b^	37.5 ± 0.3	36.8 ± 0.3^c^
Fetal weight (g)	3767 ± 297	3003 ± 301^a^	3381 ± 287	2683 ± 310^b^	3024 ± 297	2303 ± 306^c^
S/D	2.99 ± 0.22	3.55 ± 0.21^a^	3.63 ± 0.28	4.26 ± 0.25^b^	4.19 ± 0.28	4.85 ± 0.27^c^
FGR	2 (8%)	7 (28%)^a^	5 (23.8%)	10 (47.6%)^b^	6 (35.3%)	13 (76.5%)^c^

*BMI* Body Mass Index, *SBP* Systolic Blood Pressure, *DBP* Diastolic Blood Pressure, *PLT* Platelet count, *S-Cr* Serum Creatinine, *AST* Aspartate Transaminase, *ALT* Alanine Transaminase, *LDH* Lactate Dehydrogenase, *S/D* Fetal umbilical artery Systolic to Diastolic ratio, *FGR* Fetal Growth Restriction

The measurement data were expressed as mean ± standard deviation. One-way ANOVA or Chi-square test was used for comparisons between groups.

^a^ Represented in the HG group, miR200a-3p high EP group compared with the low EP group, *P* < 0.05;

^b^ Represented in the MP group, miR200a-3p high EP group compared with the low EP group, *P* < 0.05;

^c^ Represented in the SP group, miR200a-3p high EP group compared with the low EP group, *P* < 0.05

### High miR-200a-3p expression was associated with increased adverse pregnancy outcomes

**Fig. 4:**
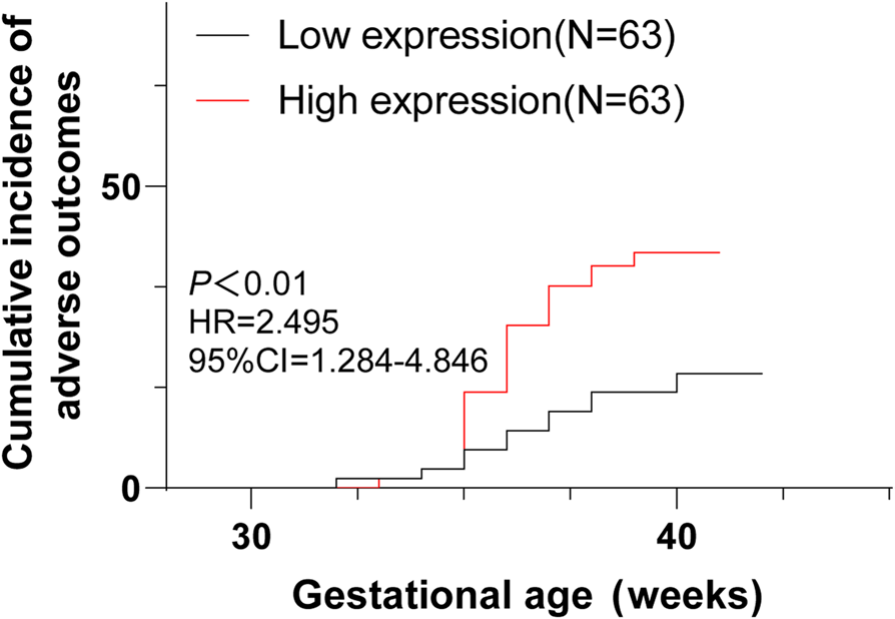
Cumulative incidence of adverse pregnancy outcomes in HDCP pregnant women. The effect of miR-200a-3p level on pregnancy outcome of HDCP patients was analyzed using the Kaplan–Meier method

According to the median value of miR-200a-3p expression, the HDCP patients were assigned to low expression group (low EP) and high expression group (high EP), and then the incidence of adverse pregnancy outcomes of the 2 groups was compared. There were significant differences in the prognosis between the 2 groups (Table 4, χ^2^ = 6.7, *P* < 0.01). The incidence of adverse pregnancy outcomes of the low EP group was 17.5%, while that of the high EP group was 38.1%. Kaplan–Meier analysis demonstrated that the curve of the high EP group was shifted to the left (Fig. 4, *P* < 0.01), indicating that at the same gestational age, the cumulative incidence of adverse pregnancy outcomes was increased in the high EP group. These results suggested that increased expression of miR-200a-3p was associated with poor pregnancy outcomes.

**Table 4:** Delivery status of HDCP patients with different miR-200a-3p expressions

	Adverse outcome	Normal delivery	Total
*miR-200a-3p* low EP group	11	52	63
*miR-200a-3p* high EP group	24	39	63
Total	35	91	126

## Discussion

Hypertension is a commonly observed disorder during pregnancy, which complicates up to 10% of the pregnancies in the women at childbearing age all over the world [12]. HDCP remain to be a leading cause of maternal mortality related to pregnancy [4]. Evidence has shown that miR-200a-3p plays an essential role in HDCP [10]. This study found that high expression of miR-200a-3p could assist the diagnosis of HDCP and predict adverse pregnancy outcomes.

Various miRNAs have been identified to be the potential early biomarkers for HDCP [13, 14]. miR-200 family has long been identified to be closely related to preeclampsia and is upregulated in placenta and plasma of the preeclampsia patients [15]. However, the expression and the diagnostic value of miR-200a-3p in HDCP still remained unclear. Our results showed that the expression of miR-200a-3p in the serum of the normal pregnant women was 1.005 ± 0.128 and that of the HDCP patients was 1.655 ± 0.379. The expression of miR-200a was gradually increased in the placenta of patients with hypertension and mild or severe preeclampsia [10]. In conclusion, high expression of miR-200a-3p may differentiate normal women from HDCP pregnant women. In order to further study the expression of miR-200a-3p in the clinical diagnosis of HDCP, we plotted the ROC curve. Our results demonstrated that the AUC was 0.9475 and the cut off value was 1.201 with the sensitivity of 87.3% and the specificity of 96.0%. In brief, miR-200a-3p level > 1.201 in the serum could assist diagnosis of HDCP. A recent study suggested that miR-200a could be explored as a promising biomarker and therapeutic target for preeclampsia [16]. Overall, our results showed the diagnostic value of serum miR-200a-3p in HDCP for the first time.

To further study whether miR-200a-3p was associated with the severity of HDCP, we allocated the HDCP patients to GH, MP and SP patients and analyzed the expression of miR-200a-3p in HDCP subgroups using the ROC curve. Our results showed that serum miR-200a-3p expression was upregulated in GH patients, its AUC = 0.9094 and the cut off value was 1.145, with 82.0% sensitivity and 88.0% specificity; compared with GH patients, serum miR-200a-3p expression was upregulated in MP patients, its AUC = 0.8126 and the cut off value was 1.541, with 73.8% sensitivity and 76.0% specificity; compared with MP patients, serum miR-200a-3p expression was upregulated in SP patients, its AUC = 0.7367 and the cut off value was 1.866, with 64.7% sensitivity and 76.2% specificity. Collectively, serum miR-200a-3p expression in GH, MP and SP was increased with the aggravation of the disease. miR-200a-3p might be a potential biomarker for the classification of HDCP severity.

Preeclampsia is characterized by proteinuria and high blood pressure [17]. AST, ALT and LDH are the clinical indicators for preeclampsia [18]. Our results demonstrated that SBP, DBP, S-Cr, AST, ALT, LDH and 24-h urine protein were increased and were increased as disease aggravation, PLT was decreased and was decreased as disease aggravation. It is consistent that LDH level was elevated in HDCP [19]. Furthermore, we allocated GH, MP and SP patients to patients with high miR200a-3p expression and low miR200a-3p expression, and analyzed the correlation between miR-200a-3p and clinical indicators of the HDCP patients. Compared with patients with low miR-200a-3p expression, BMI, SBP, DBP and AST were increased, the gestational week was advanced, fetal weight was decreased, the S/D value was increased, and the incidence of FGR was increased in patients with high miR-200a-3p expression; in MP patients, compared with patients with low miR-200a-3p expression, BMI, SBP, DBP, AST and 24-h urinary protein were increased, the gestational week was advanced, fetal weight was decreased, the S/D value was increased, and the incidence of FGR was increased in patients with high miR-200a-3p expression; in SP patients, compared with patients with low miR-200a-3p expression, BMI, SBP, DBP, S-Cr, AST, ALT, LDH and 24-h urinary protein were increased and PLT was decreased, the gestational week was advanced, fetal weight was decreased, the S/D value was increased, and the incidence of FGR was increased in patients with high miR-200a-3p expression. To date, there is no domestic and foreign report at present on the correlation between miR-200a-3p expression and clinical indicators in the serum of HDCP patients. Our results indicated that miR-200a-3p had a close correlation with clinical indicators of HDCP patients for the first time.

In addition, a variety of miRNAs are closely related to adverse pregnancy outcomes [20]. We analyzed the incidence of adverse pregnancy outcomes of the HDCP patients with high and low miR-200a-3p expression. Our results showed that the incidence of adverse pregnancy outcomes of patients with low miR-200a-3p expression was 17.5%, while that of the patients with high miR-200a-3p expression was 38.1%. The results of Kaplan–Meier analysis indicated that the curve of the high miR-200a-3p expression patients was shifted to the left, which suggested that the cumulative incidence of adverse pregnancy outcomes was increased in patients with high miR-200a-3p expression at same gestational age. Alterations in myometrial expression of miR-200 family may play a role in the induction of preterm labor [21, 22]. In conclusion, our study initially revealed that increased expression of miR-200a-3p was related to poor pregnancy outcomes.

In summary, as a prospective study, this study detected the expression of miR-200a-3p in serum of HDCP patients and explored the role of miR-200a-3p expression in the diagnosis of HDCP using ROC curve for the first time, and supported that high expression of miR-200a-3p could assist the diagnosis of HDCP and predict adverse pregnancy outcomes, which provided a new entry point for clinical condition judgment and adverse outcome prediction of HDCP. However, the number of cases and events included in this study is small. It is necessary to further expand the sample size and carry out a multi-center study to further clarify the diagnostic and prognostic ability of miR-200a-3p. The correlation analysis of gestational age and miRNA expression was not studied. The role of miR-200a-3p in the pathogenesis of HDCP remains unclear, either. In addition, it is principal to determine the expression of serum miR-200a-3p in the early or middle pregnancy, and study its diagnostic and prognostic value in the early stage of the disease and explore the mechanism of miR-200a-3p in the pathogenesis of HDCP and the target of miR-200a-3p at protein levels.

## Data Availability

All the data generated or analyzed during this study are included in this published article.
